# The effect of web 2.0 based technology applications on speaking skills and speaking anxiety in teaching Turkish as a foreign language: Voki example

**DOI:** 10.3389/fpsyg.2023.1183037

**Published:** 2023-06-14

**Authors:** Elif Aktaş

**Affiliations:** Department of Turkish and Social Sciences Education, Education Faculty, Alanya Alaaddin Keykubat University, Antalya, Türkiye

**Keywords:** Voki, speaking anxiety, speaking skill, teaching Turkish as a foreign language, web 2.0 tools

## Abstract

The aim of this study is to examine the effect of Voki, one of the web 2.0-based technology applications, on Turkish learners’ speaking skills and speaking anxiety. In the study, exploratory sequential design, one of the mixed design types in which quantitative and qualitative approaches are used together, was adopted. The study group of the research consisted of 61 students at A2 level (31 experimental group, 30 control group) who learn Turkish as a foreign language at the Turkish Language Teaching Center of a university located in the south of Turkey. Speaking Anxiety Scale and Speaking Skill Assessment Form were used as data collection tools. During the 6-week intervention, the experimental group used Voki in speaking lessons, while the control group did not use any technology-based web 2.0 tool. Descriptive statistics, chi-square analysis, dependent and independent groups *t*-test were used to analyze the quantitative data collected in the study. Descriptive analysis and content analysis were used to analyze the qualitative data collected through a semi-structured interview form. As a result of the study, it was determined that the Voki application improved the speaking skills of the students in the experimental group and reduced their speaking anxiety. It was also determined that the students in the experimental group expressed positive opinions about the application. Therefore, the use of Voki application in speaking activities in foreign language teaching is recommended.

## 1. Introduction

Technology is rapidly being integrated into educational activities as it is in every field. So much so that technology has become an integral part of the learning process in the modern world (Aikina and Zubkova, 2015). Today, there are many web 2.0 applications that will contribute to the integration of the learning-teaching process with technology (Durusoy, 2011). Web 2.0 tools, which enable the creation of online digital content, are defined as a name given to all websites that allow users to create, modify and share Internet content (Cambridge Dictionary, 2022). With the existence of Web 2.0 tools, the teaching-learning process no longer takes place only in classrooms, but anywhere and at any time. In this regard, the nature of educational activities today has changed from being teacher-centered to interactive environments where there is collaboration between teachers and students (Manty et al., 2017).

With Web 2.0 tools, students can improve their basic language skills such as listening, speaking, reading and writing by collaborating and interacting in a natural learning environment (Arıkan and Özel, 2015; Baş and Yıldırım, 2018). Web 2.0 tools create a fun and interesting learning environment by allowing students to interact with each other (İnce and Akdemir, 2013; Agravat and Raval, 2015). In this respect, it is very effective in developing language skills, especially speaking skills, especially in the second and foreign language learning process (Yeşilbağ and Korkmaz, 2021).

One of the web 2.0 applications for developing basic language skills in foreign language teaching is Voki (Yeşilbağ and Korkmaz, 2021). Voki, a podcast application, is a creative web 2.0 tool that can be used especially for practicing speaking in foreign language classes. With Voki, it is possible to create cartoon characters called avatars in online environments and vocalize written texts with these characters (Agravat and Raval, 2015). Voki is a web-based application that can be used on computers, tablets and smartphones. In the application consisting of 4 parts, Voki, Voki Classroom, Voki Presenter and Voki Hangout, it is possible to make voice-overs in more than 30 languages. With Voki, which is a useful application for students who are afraid of speaking in the classroom environment, students can record their voices and share voice messages. Shaping the avatar created through the application in the desired way and making it speak creates a fun learning environment (Lornsen, 2010). Voki can be effective in improving students’ speaking, listening and writing skills and increasing their motivation and creativity. In addition, it is also possible for students who have pronunciation problems in foreign language learning to improve themselves in speaking fluently in extracurricular activities and to improve their writing skills by writing the text they will vocalize (Aikina and Zubkova, 2015; Baş and Yıldırım, 2018).

Language teaching is based on four basic skills: listening, speaking, reading and writing. Among these skills, writing and speaking are the last and most difficult skills to acquire. Speaking, which is one of the most used skills in daily life, has a greater role than other skills in communicating with each other (Tanrıkulu and Şıhanlıoğlu, 2019). According to the communicative approach that has shaped foreign language teaching for a long time, the main purpose of language learning is to speak, that is, to communicate (Keser, 2018). As a matter of fact, speaking is one of the main indicators of knowing a foreign language. Because if a person cannot speak in a foreign language, he/she is not considered to know that language completely (Çelik, 2019). In foreign language teaching, it is essential to teach and learn the language to communicate. In addition, one of the most important goals of teaching is for learners to be able to speak the target language comprehensibly (Demirel, 2014).

The teaching of speaking skill is multifaceted. In foreign language teaching, speaking skill is handled as skill acquisition and use activities. At the skill acquisition stage, the student learns about grammar, vocabulary and pronunciation and applies them in classroom speaking activities. This is followed by activities in which the learner uses the language outside the classroom, in real contexts. Skill acquisition activities prepare the learner to use the language in real life. At this stage, the learner is first given mechanical speaking activities such as repetition exercises. Then they move on to communication-oriented exercises involving real-life dialogs (Rivers and Temperley, 1978). In teaching speaking skills, real-life based activities can be done in and out of the classroom with technology applications that encourage creativity such as Voki. Voki is a fun and effective application used especially to improve foreign language learners’ pronunciation skills (Dona et al., 2014) and to practice listening, reading and writing (Mirtschin, 2010; Aikina and Zubkova, 2015).

It is well known that some students are shy about expressing their ideas in a foreign language. Such students need time or courage to put their ideas into words. One of the biggest problems in the difficult and late acquisition of speaking skills in foreign language teaching is students’ fear of making mistakes. Some web 2.0 tools provide opportunities for students to improve their speaking practice in a foreign language. Voki is an instructive web tool that can be used to overcome or reduce the fear of speaking in a foreign language. In this direction, Voki, which provides the opportunity to create an online character and share their thoughts comfortably through this character, helps students overcome their shyness and overcome the anxiety of speaking in a foreign language (Yalçın, 2020). Voki, which allows students to speak without showing their own faces while recording, is also effective in developing fluency and pronunciation skills and learning the accent of the target language (Aikina and Zubkova, 2015). Voki develops not only speaking but also writing skills. Because the student writes the text to be voiced by the cartoon character called avatar. Accordingly, when the student writes the part that needs to be vocalized incorrectly, the cartoon character does not vocalize it correctly. In addition, thanks to Voki, students have the opportunity to use their listening skills effectively while practicing writing and speaking.

When the related literature is examined, there are studies that mention the use of Voki in foreign language teaching and its advantages. For example, Ramdani (2018) found that thanks to Voki in teaching English as a foreign language, students were exposed to speaking English outside the classroom, they were able to cooperate among themselves and with the teacher, they felt comfortable while learning the language, and their motivation to speak increased. Yeşilbağ and Korkmaz (2021) found that students’ speaking skills improved with Voki in teaching English as a foreign language, but this practice did not change their attitude toward the course. Zargaryan (2012) also concluded that Voki is especially useful in developing speaking skills in a foreign language. Manty et al. (2017) emphasized that Voki used in foreign language teaching helps students improve their speaking skills in terms of fluency and pronunciation. Picardo (2009) stated that Voki in foreign language classes contributed positively to students’ grammar learning and helped them to have a larger vocabulary. He also emphasized that thanks to Voki’s ability to make voice recordings at home, students became more confident in speaking Spanish and their participation in the lessons increased. Setyawati and Azizaturrohmah (2016) stated that Voki is an effective learning tool that enriches the teaching process for teachers and enables students to practice speaking independently of time and place and increases their motivation. The fun and engaging aspects of applications such as Voki can also be useful in teaching basic language skills (Yazar, 2019). Özdemir (2017) and Baş and Yıldırım (2018) also stated that Voki has a positive effect on listening, speaking and writing skills, pronunciation, motivation and creativity in teaching Turkish as a foreign language. Nguyen Thi Kieu and Nguyen (2021) found that Voki improved high school students’ foreign language speaking skills and that students were satisfied with this practice, their self-confidence increased, and their shyness and uneasiness decreased.

## 2. Problem and research questions

In this study, the effect of Web 2.0-based Voki application on speaking skills and speaking anxiety in teaching Turkish as a foreign language was examined. In addition, the opinions of the students in the experimental group about the application were taken. The main problem of the research is; “What is the effect of Web 2.0-based Voki application on speaking skills and speaking anxiety in teaching Turkish as a foreign language?” is in the form of. The sub-problems are as follows:

- Is there a significant difference between the mean test scores of the students in the experimental group using Voki application and the control group using the current curriculum?
- Is there a significant difference between the scores for speaking anxiety of the students in the experimental group in which the Voki application was used and the control group in which the current curriculum was used?
- What are their views on the practice of the students in the experimental group in which Voki application was used?

## 3. Method

### 3.1. The research design

In the study, the exploratory sequential design, one of the mixed research designs in which qualitative and quantitative research techniques are used together, was preferred. Combining the strengths and weaknesses of two or more research methods reduces the possibility of error (Johnson and Christensen, 2004). In this design, quantitative data are first collected and analyzed. Then qualitative data are used to explain the quantitative data in detail (Creswell, 2014). In this study, quantitative data were first collected and analyzed, and then the quantitative data were supported by semi-structured interviews with the students in the experimental group.

In the quantitative dimension of this study, a pretest-posttest control group experimental design was used. In this model, there are two groups (one experimental and one control) determined by random assignment and measurements are carried out before and after the experiment in both groups (Karasar, 2012). The experimental group used the web 2.0-based Voki application during the speaking lessons, while the control group did not use Voki or any other web 2.0 tool. In the qualitative dimension of the research, which was designed as a case study, data were collected and analyzed through interviews aiming to determine the views of the students in the experimental group on the Voki application.

Chi-square test was used to compare the descriptive characteristics of the students in the experimental and control groups. *T*-test for independent groups and *T*-test for dependent groups were used to compare the pre-test and post-test values of the students in the experimental and control groups before and after the application within and between groups. Qualitative data were analyzed using descriptive analysis and content analysis.

### 3.2. Participants

In the spring semester of the 2021–2022 academic year, 31 students at A2 level learning Turkish at the Turkish Language Teaching Center (TÖMER) of a university in southern Turkey were assigned as the experimental group and 30 students were assigned as the control group. The characteristics of the participants determined by random sampling method are as follows:

In the experimental group, 10 of the students were female and 21 were male; in the control group, 10 of the students were female and 20 were male. Of the students in the experimental group, 20 were between the ages of 18–23, 9 were between the ages of 24–29, and 2 were 30 years and older. In the control group, 27 of the students were between the ages of 18–23 and 3 were between the ages of 24–29. The countries of the students in the experimental group were Afghanistan (*f* = 13), Yemen (*f* = 11), Pakistan (*f* = 3), Democratic Congo (*f* = 2), Egypt (*f* = 2). The countries of the students in the control group are Somalia (*f* = 12), Indonesia (*f* = 4), Kazakhstan (*f* = 3), Iran (*f* = 2), Uzbekistan (*f* = 2), Syria (*f* = 2), Jordan (*f* = 2) and Tanzania.

The sample selection regarding the qualitative dimension of the research was limited to 30 students in the experimental group. Since it was aimed to reveal the opinions and thoughts of the students about the Voki application and to make an in-depth description of this situation, the qualitative dimension of the study was carried out only with the experimental group students (*f* = 30) in which the Voki applications were carried out. In the qualitative dimension of the study, 10 of the participants were female and 20 were male. Twenty of the participants were between the ages of 18–23 and 10 of them were between the ages of 24–29 and their countries were Afghanistan (*f* = 12), Yemen (*f* = 11), Pakistan (*f* = 3), Democratic Congo (*f* = 2), Egypt (*f* = 2).

### 3.3. Data collection tools

In the study, Speaking Skill Assessment Form and Speaking Anxiety Scale were used to obtain quantitative data. Semi-structured Interview Form was used to collect qualitative data.

#### 3.3.1. Speaking skills assessment form

The Speaking Skill Assessment Form developed by Günaydın (2020) was used to measure students’ Turkish speaking skills. This form was used as a pre-test to evaluate the students’ recorded speech before the experimental process. At the end of the six-week experimental process, the students’ speech recordings were again evaluated with this form. Consisting of 17 items, the highest score that can be obtained from the form is 85 and the lowest score is 0. According to the 6-point Likert-type rating scale, there are 6 options ranging from 0 to 5. The scores obtained from the scale vary between 0 and 5.

Before the Speaking Skill Assessment Scale was administered, open-ended questions were created by the researchers within the scope of the speaking topics of the Turkish as a foreign language course curriculum at the A2 level in order to determine the speaking levels of the students. The opinions of 3 field experts were utilized in determining the speech topics. One of the determined 10 open-ended questions was directed to the experimental and control group students before and after the experimental procedure, and the speaking skills of the students were evaluated in accordance with the criteria in the aforementioned form.

For rater reliability, the correlation coefficient between the pre-test and post-test assessment results of the two researchers was calculated as 0.92 and 0.97. It is stated that the correlation value is low when it is less than 0.30, moderate when it is between 0.30 and 0.70, and high when it is greater than 0.70 (Büyüköztürk et al., 2015). In this case, it can be said that the agreement between the two raters is at a good level.

#### 3.3.2. Speech anxiety scale

In the study, the scale developed by Woodrow (2006) under the name “The second language speaking anxiety scale” and adapted into Turkish by Melanlıoğlu and Demir (2013) was used to measure the speaking anxiety of students learning Turkish as a foreign language. Consisting of 12 items, the scale has 2 sub-factors: in-class speaking anxiety (1, 3, 4, 5, 6) and out-of-class speaking anxiety (2, 7, 8, 9, 10, 11, 12). According to the 5-point Likert-type rating scale, there are 5 options ranging from “I do not worry at all (1)” to “I worry a lot (5).” The scores obtained from the scale vary between 1 and 5. Accordingly, those who score 1.00–1.79 on the scale are evaluated as “very low”; those who score 1.80–2.59 as “low”; those who score 2.60–3.39 as “medium”; those who score 3.40–4.19 as “high”; and those who score 4.20–5.00 as “very high.” As the score obtained from the scale decreases, the anxiety level of the students decreases, and as the score increases, the anxiety level of the students increases.

Cronbach’s alpha internal consistency coefficient was calculated to determine the reliability of the scale. The alpha coefficients calculated for the internal consistency of the scale, which consists of 2 factors: in-class and out-of-class speaking anxiety, vary between 0.55 and 0.72. Accordingly, the scale was found to be reliable as in-class speaking anxiety 0.90, out-of-class speaking anxiety 0.93 and total 0.91. The test–retest reliability coefficient for the whole scale was found to be 0.90. The Cronbach-alpha value calculated for the whole scale in this study was 0.87.

#### 3.3.3. Semi-structured interview form

The interview technique was used to obtain the opinions of the students in the experimental group after the application. Interview is a reciprocal, interactive and exploratory process that is conducted in the form of question-answer in line with a purpose and where individuals’ opinions on various issues are taken (Patton, 2002; Glesne, 2013). The interview technique allows participants to explain their ideas in more depth and to evaluate the situation from the participant’s perspective (Cohen et al., 2011).

In this research process, semi-structured interviews were conducted with the students in the experimental group in order to determine their views on the application in full and in depth. In this direction, an interview form consisting of three open-ended questions was prepared by reviewing the relevant literature. In order to ensure the content validity of the form, two experts from the field of measurement and evaluation and two experts from the field of Turkish education were consulted. In the form, students were asked to explain whether they were satisfied with the Voki creation process (and which stages they were satisfied with), whether they had difficulties in this process (and at what stage), the benefits of the Voki application for them, with their justifications.

In the final version of the semi-structured interview form, students were asked the following three pre-prepared questions:

- What is your level of satisfaction with the voki creation process? At which stage were you more satisfied? Explain with justifications.
- What difficulties did you experience during the voki creation process? At which stage did you have more difficulties? Explain with justifications.
- What were the benefits of the voki application for you? Explain with justifications.

### 3.4. Process

Implementation, it was conducted by one of the researchers in the Turkish for foreigners course conducted for 6 weeks in the spring semester of the 2021–2022 academic year. Before the implementation, the scales to be used in the study (Speaking Skill Assessment Form and Speaking Anxiety Scale) were administered as a pre-test to determine the current status of the students in the experimental and control groups. The 1 week before the application covers the preparation process for the actual application. During this process, presentations introducing the Voki application were made to the experimental group and sample applications were asked from them. In addition, introducing the application web address and documents were given to the students. Since these preliminary preparations contributed positively to the Voki creation process, the implementation was carried out easily. During the implementation process, the students in the experimental group presented and evaluated the Voki samples they prepared in the lesson. In the experimental group, speaking lessons were conducted for a total of 24 h, 4 h a week for 6 weeks. In the control group, where the current curriculum was carried out, the course was conducted under the guidance of the instructor without using web 2.0 tools. After 6 weeks of implementation, the Speaking Skills Assessment Form and Anxiety Scale were administered again as a post-test. Finally, semi-structured interviews were conducted with the students in the experimental group (*f* = 30) on a voluntary basis.

**Figure 1:**
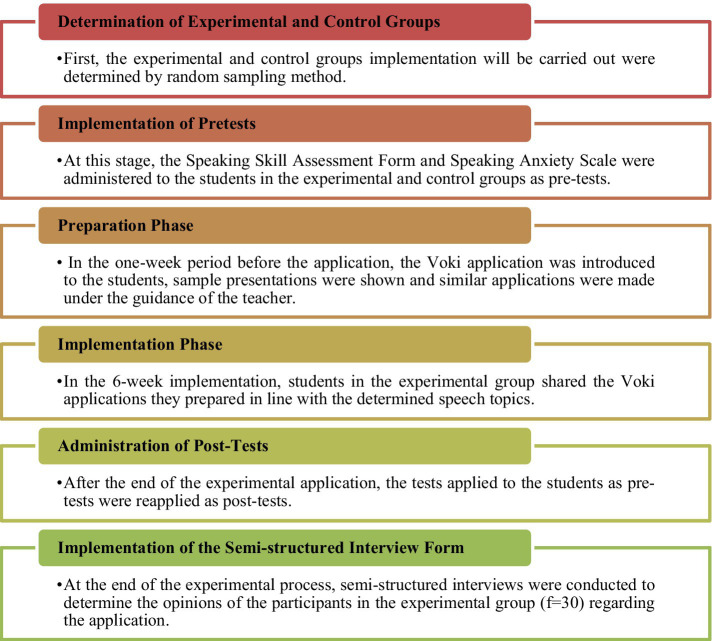
Steps of the experimental process.

The steps of the experimental process are shown in Figure 1.

#### 3.4.1. The process conducted in the experimental and control groups

In line with the 6-week lesson plan, the speaking topics in the curriculum were prepared by the students using the Voki application. Throughout the experimental process, students created at least 6 Voki in 4 h of speaking lessons per week and shared their Voki with their teachers and friends via e-mail.

**First week:** In the ‘Gezelim Görelim’ unit, students acquired prior knowledge about speaking in grammar, reading and listening lessons. In the 4-h speaking lesson, students were asked to describe their friends using the words they had just learned with the Voki application. Visual selections (such as avatars/characters, clothes, places, etc.) were made by the students in the lesson under the guidance of the teacher. The students shared the Voki they organized in line with the feedback they received with the class group via e-mail the following week.

**Second week:** In the “Haberiniz Olsun” unit, students vocalized the text they wrote about famous people in the writing lesson using the Voki application. During the Voki creation phase, the teacher guided the students in choosing visuals such as avatar/character, clothing, location, etc.

**Third week:** In the “Neler Olacak” unit, students had an impromptu conversation about dreams using the Voki app.

**Fourth week:** In the “Evvel Zaman İçinde” unit, students visualized and vocalized the writings they wrote about their future plans under the guidance of their teachers using the Voki application.

**Fifth week:** In the “Ne Olur Ne Olmaz” unit, students prepared their essays about natural disasters using the Voki application.

**Sixth week:** In the “Neler Yapabilirsiniz?” unit, students prepared a speech about their travel plans with the Voki application, and completed their presentations with appropriate avatar and visual selections.

During the implementation, the same units were taught in the control group in line with the existing curriculum. In the control group, a communicative approach that emphasizes interaction and communication was preferred. Students presented the current speaking topics with dialog, discussion, prepared and unprepared speaking techniques. Smart board, textbook, videos and PowerPoint presentations were used as tools and materials. In addition, no web 2.0 tool including Voki was preferred in the control group.

### 3.5. Data analysis

The statistical analyses used in the analysis of quantitative data in the study are shown in below:

Chi-square test was used to compare the descriptive characteristics of the students in the experimental and control groups. *T*-test for independent groups and *T*-test for dependent groups were used to compare the pre-test and post-test values of the students in the experimental and control groups before and after the application within and between groups.

Skewnes-Kurtosis analyzes were performed to determine the conformity of the data collected from the students in the experimental and control groups to the normal distribution. Skewnes-Kurtosis values between +1.96 and −1.96 are accepted as normal distribution (George and Mallery, 2010). In this study, it can be said that Skewnes-Kurtosis values for all test scores showed a normal distribution. Therefore, parametric analyses were applied in the data analysis since the number of people in the group exceeded 30.

The qualitative data of the study were analyzed by descriptive analysis and content analysis. In this direction, the opinions of the experimental group students were grouped under certain themes and supported with direct quotations. To ensure the accuracy of the participants’ responses, expert interpretations of the themes were cross-checked (Creswell and Plano Clark, 2018): Accordingly, 80% of the data were independently coded by another field expert (Turkish language education). Cohen’s Kappa statistic was used to compare and evaluate the different codings created by independent coders. Accordingly, 0.80 and above is considered to indicate very good agreement and acceptable reliability. Inter-rater reliability was then calculated as a percentage and found to be high (94%). Two language trainers working on a similar topic also examined the emerging themes. Minor disagreements were discussed and consensus was reached.

## 4. Findings

### 4.1. Findings related to quantitative data

Information about the comparison of the students in the experimental and control groups in terms of their identifying characteristics is given in Table 1.

**Table 1:** Comparison of students in the experimental and control groups in terms of their identifying characteristics.

		Experiment		Control		*χ* ^2^	*p*
		*n*	%	*n*	%		
Age	18–23 years old	20	64.5	27	90.0	6.028	0.049
	24–29 years old	9	29.0	3	10.0		
	30 years and older	2	6.5	0	0.0		
Gender	Woman	10	32.3	10	33.3	0.008	0.929
	Male	21	67.7	20	66.7		

According to the age characteristics in Table 1, 64.5% of the students in the experimental group were 18–23 years old, 29% were 24–29 years old, 6.5% were 30 years old and above; 90% of the students in the control group were 18–23 years old and 10% were 24–29 years old. The difference between the groups was statistically significant at *p* < 0.05 significance level. It is seen that the students in the control group are younger than those in the experimental group.

Table 1 shows that 32.3% of the students in the experimental group were female and 67.7% were male, while 32.2% of the students in the control group were female and 67.7% were male. The difference between the groups was found to be statistically insignificant at *p* > 0.05 significance level.

The *t*-test results of the comparison of the pretest scores of the students in the experimental and control groups from the Speaking Skill Assessment Form, Out-of-Class and In-Class Speaking Anxiety Scale are given in Table 2.

**Table 2:** Comparison of the pre-test scores of the students in the experimental and control groups on the speaking skill assessment form, out-of-class and in-class speaking anxiety scale after the intervention.

Pre-test		*N*	$\bar{X}$	S.s	*t*	*p*
Speaking skills assessment form	Experimental group	31	74.03	3.755	10.476	0.000
	Control group	30	64.57	3.277		
Out-of-class speaking anxiety scale	Experimental group	31	8.97	2.273	−13.003	0.000
	Control group	30	15.90	1.863		
Classroom speaking anxiety scale	Experimental group	31	15.61	3.030	−10.893	0.000
	Control group	30	23.80	2.833		

Table 2 shows that the difference between the pretest scores of the students in the experimental and control groups on the Speaking Skill Assessment Form was not significant at the *p* > 0.05 significance level (*t* = 0.383 *p* = 0.703).

According to Table 2, it is seen that the difference between the pretest scores of the students in the experimental and control groups from the Out-of-Class Speaking Anxiety Scale is not significant at the *p* > 0.05 level of significance (*t* = −1.757, *p* = 0.084). It is seen that the difference between the pre-test scores of the same students from the In-Class Speaking Anxiety Scale is insignificant at the *p* > 0.05 significance level (*t* = −1.706, *p* = 0.093).

As a result, it can be said that there was no difference between the students in the experimental and control groups in terms of their pretest scores on the Speaking Skill Assessment Form, Out-of-Class and In-Class Speaking Anxiety Scale.

The *t*-test results for the comparison of the post-test scores of the students in the experimental and control groups from the Speaking Skill Assessment Form, Out-of-Class and In-Class Speaking Anxiety Scale are given in Table 3.

When Table 3 is examined, it is seen that the difference between the post-test scores of the students in the experimental and control groups from the Speaking Skills Assessment Form is significant at *p* < 0.05 significance level (*t* = 10.476, *p* = 0.000). According to Table 3, the average posttest score of the students in the experimental group on the Speaking Skills Assessment Form was 74.03. This result is higher than the mean post-test score of 64.57 in the control group. As a result, it can be said that the Voki application used in the experimental group was effective on the speaking skills of Turkish as a foreign language learners.

**Table 3:** Comparison of speaking skill assessment form, out-of-class and in-class speaking anxiety scale pre-test scores of students in experimental and control groups.

Pre-test		*N*	$\bar{X}$	S.s	*t*	*p*
Speaking skills assessment form	Experimental group	31	63.10	2.271	0.383	0.703
	Control group	30	62.87	2.417		
Out-of-class speaking anxiety scale	Experimental group	31	14.29	2.795	−1.757	0.084
	Control group	30	15.33	1.688		
Classroom speaking anxiety scale	Experimental group	31	24.94	3.316	1.706	0.093
	Control group	30	23.60	2.762		

It is seen that the difference between the posttest scores of the students in the experimental and control groups on the Out-of-Class Speaking Anxiety Scale is significant at the *p* < 0.05 significance level (*t* = −13.003, *p* = 0.000). According to Table 3, the mean posttest score of the experimental group on the Out-of-Class Speaking Anxiety Scale was 8.97. This result is smaller than the post-test mean score of 15.90 in the control group. As a result, it can be said that the Voki application used in the experimental group reduced out-of-class speaking anxiety.

It is seen that the difference between the post-test scores of the students in the experimental and control groups on the Classroom Speaking Anxiety Scale is significant at *p* < 0.05 level of significance (*t* = −10.893, *p* = 0.000). When Table 3 is examined, it is seen that the mean posttest score of the experimental group on the Classroom Speaking Anxiety Scale is 15.61, which is lower than the mean posttest score of the control group, which is 23.80. As a result, it can be said that the Voki application used in the experimental group reduced classroom speaking anxiety.

The comparison of the pre-test and post-test scores of the students in the experimental group from the Speaking Skill Assessment Form, Out-of-Class and In-Class Speaking Anxiety Scale before and after the application is given in Table 4.

**Table 4:** Comparison of the pre-test-post-test score averages of the students in the control group in the speaking skills assessment form, out-of-class and in-class speaking anxiety scale.

		*N*	$\bar{X}$	S.s	*t*	*p*
Speaking skills assessment form	Pre-test	30	62.87	2.417	−3.635	0.001
	Post test	30	64.57	3.277		
Out-of-class speaking anxiety scale	Pre-test	30	15.33	1.688	−3.084	0.004
	Post test	30	15.90	1.863		
Classroom speaking anxiety scale	Pre-test	30	23.60	2.762	0.947	0.351
	Post test	30	23.80	2.833		

When Table 4 is examined, the *t*-test analysis of the difference between the pre-test and post-test mean scores of the students in the experimental group on the Speaking Skill Assessment Form, Out-of-Class and In-Class Speaking Anxiety Scale before and after the application was found to be significant in favor of the post-test at *p* < 0.05 level of significance.

It was determined that the Voki application was effective on the Speaking Skill Assessment Form, Out-of-Class and In-Class Speaking Anxiety Scale scores of the students in the experimental group. With this result, it can be said that Voki improves the speaking skills of Turkish as a foreign language learners and reduces their speaking anxiety in and out of the classroom.

The comparison of the pre-test and post-test scores of the students in the control group from the Speaking Skill Assessment Form, Out-of-Class and In-Class Speaking Anxiety Scale before and after the application is given in Table 5.

**Table 5:** Comparison of the experimental group students’ speaking skill assessment form, out-of-class and in-class speaking anxiety scale pre-test-post-test score averages.

		*N*	$\bar{X}$	*S.s*	*t*	*p*
Speaking skills assessment form	Pre-test	31	63.10	2.271	−19.587	0.000
	Post test	31	74.03	3.755		
Out-of-class speaking anxiety scale	Pre-test	31	14.29	2.795	15.021	0.000
	Post test	31	8.97	2.273		
Classroom speaking anxiety scale	Pre-test	31	24.94	3.316	19.771	0.000
	Post test	31	15.61	3.030		

When Table 5 is examined, the *t*-test analysis of the difference between the pre-test and post-test mean scores of the students in the control group in the Speaking Skills Assessment Form and Out-of-Class Speaking Anxiety Scale before and after the application was found to be significant in favor of the post-test at *p* < 0.05 level of significance.

As a result, it was seen that the traditional implementation was effective on the Speaking Skill Assessment Form and Out-of-Class Speaking Anxiety Scale scores of the students in the control group, in other words, the implemented program increased speaking skills while increasing out-of-class speaking anxiety.

As a general result, it can be said that the Voki application used in the experimental group was more effective in improving speaking skills and reducing in-class and out-of-class speaking anxiety than the traditional education applied to the control group.

### 4.2. Findings related to qualitative data

At the end of the application, a semi-structured interview form was used to support the quantitative data with qualitative data and to obtain the opinions and thoughts of the students in the experimental group about the Voki applications. The opinions obtained from the forms were first subjected to descriptive analysis and then content analysis and codes and themes were determined. The data were grouped under three headings around three questions and frequency analysis was performed (Tables 6–8):

**Table 6:** Opinions on satisfaction or dissatisfaction with the voki creation process.

Theme	Opinion/code	*f*	%
Avatar/character selection	*I had a lot of fun choosing an avatar (S2)* *I was happy to choose among various avatars (S10)*	25	83.3%
Voiceover	*I liked that they did not see me while I was doing the voice-over. (S6)* *I pronounced the words better with Voki (S3)*	18	60%
Visualization	*I had a lot of fun choosing a place while creating a voki. (S3)* *It was fun to choose the character’s face and clothes (S10)*	13	43.3%
Preparing/writing a speech	*It was a nice experience to prepare a speaking position. (S14)* *It was good to write down what I was going to talk about. (S10)*	2	6.6%

**Table 7:** Opinions on which stage of the voki creation process they had difficulties.

Theme	Opinion/code	*f*	%
Avatar/character creation	*I could not use some characters because they are paid (S5)* *Free Voki was very limited, it was difficult for me (S12)*	16	53.3%
Visualization	*I had difficulty in finding the visuals I wanted (S1)* *I had difficulty in selecting them because there were so many different visuals. (S10)*	13	43.3%
Voiceover	*The time given to make the avatar talk was short. For this reason, I created an avatar again. (S11)* *Since the time was limited (60 s), I could not vocalize as I wanted. (S11)*	12	40%

**Table 8:** Opinions on the benefits of the voki application.

Theme	Opinion/code	*f*	%
Speaking skills	*It helped me express myself better. (S6)* *It helped me pronounce words more accurately. (S14)*	25	83.3%
Speech anxiety	*I feel less anxious when I speak (S3)* *I speak more comfortably. (S6)*	21	70%
Motivation	*It helped me to increase my self-confidence. (S13)* *I am more free to express myself (S14)*	19	63.3%
Permanent learning	*I did research on the given topics and learned new words. (S9)* *Time and space were unlimited. In this way, I realized my mistakes in my speech and did not make them again. (S11)*	15	50%
Writing skills	*Writing my speech text improved my writing (S3)* *I learned the correct spelling of words (S5)*	13	43.3%
Listening skills	*My listening skills also improved while creating voki. (S3)* *It was nice to listen to the avatars of my friends and myself. (S8)*	2	6.6%
Ability to use technology	*In this lesson, I realized that technology can be used in a useful way. (S10)* *Thanks to these applications, my ability to use technology has increased. (S14)*	2	6.6%
No benefit	*Choosing an avatar and voicing it was childish. (S2)* *It was useless to talk in a virtual environment. (S4)* *There was a very limited content (S15)* *It was a simple application. It was not useful. (S16)* *The free version was very simple. It was not useful (S2)*	5	16.6%

At the end of the application, the students in the experimental group were asked whether they were satisfied with the Voki applications. Accordingly, the majority of the students in the experimental group (*n* = 25) stated that they were satisfied with the Voki application. According to Table 6, when the students’ opinions on which stage of the Voki creation process they were most satisfied with were examined, it was determined that 83.3% were satisfied with the avatar/character selection stage, 60% with the voiceover stage, 43.3% with the visualization stage, and 6.6% with the speech text preparation/writing stage.

Table 7 shows the opinions of the students on which stage of the Voki application they had the most difficulty. Accordingly, 53.3% of the students stated that they had difficulty at the avatar/character creation stage, 43.3% at the visualization stage, and 40% at the voiceover stage.

When Table 7 is analyzed, it is noteworthy that the difficulties students experienced with the Voki application were generally related to free membership. Students stated that they had difficulties with the free membership because of the limited visual selection and voice recording time.

When the data in Table 8 are analyzed, it is seen that the students in the experimental group generally expressed positive opinions about the Voki application. Accordingly, 83.3% of the students stated that the Voki application improved speaking skills, 70% stated that it reduced speaking anxiety, 63.3% stated that it increased motivation, 50% stated that it provided permanent learning, 43.3% stated that it improved writing skills, and 6.6% stated that it improved technology use and listening skills.

In Table 8, it was also determined that 5 students found this application childish and simple and did not find it useful because they thought it had a limited content.

## 5. Results and discussion

Within the scope of the first question of the research, a significant difference was found in favor of the experimental group in terms of speaking skills at the end of the experimental application between the experimental group where Voki application was used and the control group where the current curriculum was applied. At the end of the experimental application, while the speaking skill test results of the experimental and control groups increased, the increase in the experimental group was higher than the control group. This result shows that Voki practices have a positive effect on speaking skills. Most studies in the literature also show that Voki improves speaking skills, especially in foreign language teaching, as it enables students to actively participate in the learning process by vocalizing written texts through the avatars they choose and creates a fun learning environment (Picardo, 2009; Zargaryan, 2012; Svendsen et al., 2014; Setyawati and Azizaturrohmah, 2016; Manty et al., 2017; Özdemir, 2017; Bellés-Calvera and Bellés-Fortuño, 2018; Pereira and Silva, 2018; Ramdani, 2018; Namaziandost and Nasri, 2019; İstifanoğlu, 2020; Nguyen Thi Kieu and Nguyen, 2021; Yeşilbağ and Korkmaz, 2021).

Within the scope of the second questions of the research, a significant difference was found between the experimental group in which Voki was applied and the control group in which the current curriculum was applied in favor of the experimental group in terms of speaking anxiety scores at the end of the experimental application. Accordingly, at the end of the experimental application, it was determined that the out-of-class and in-class speaking anxiety of the experimental group students decreased. When the related literature is examined, it is found that Voki has a positive effect on student attitudes (Zargaryan, 2012; Bellés-Calvera and Bellés-Fortuño, 2018) and self-confidence development (İstifanoğlu, 2020) as well as reducing speaking anxiety (Svendsen et al., 2014; Yona and Marlina, 2014).

In this study, the opinions of the students in the experimental group about the Voki application were also taken to support the quantitative data. The third problem of the research is to determine these opinions. As a result of the experimental process in this study, the majority of the students expressed positive opinions about the Voki practices. Within the scope of the results obtained from semi-structured interviews, students stated that Voki applications improved their speaking and pronunciation skills and reduced their speaking anxiety. Students also stated that Voki made the lesson more enjoyable and fun and increased motivation by ensuring active participation in the learning process. Similar to this result, Setyawati and Azizaturrohmah (2016) found in their study that Voki practice increases speaking motivation in foreign language teaching. In the related literature, many studies have shown that Voki improves students’ speaking and communication skills, increases their desire and creativity to speak in the target language, helps them plan their oral presentations, helps them remember vocabulary and language structures better, and enables individual work (Svendsen et al., 2014; Yona and Marlina, 2014; Baş and Yıldırım, 2018; Pereira and Silva, 2018; Nguyen Thi Kieu and Nguyen, 2021). In this study, students also stated that they were satisfied with Voki because it provided a fun, enjoyable, stress-free and effective learning environment. Similarly, in some studies, students reported that Voki was an effective (Svendsen et al., 2014) and enjoyable application in providing intrinsic motivation in the foreign language learning process (Pereira and Silva, 2018).

In this study, students reported that the Voki application improved their language skills. With this application, students stated that they learned both the spelling and pronunciation of words, and that they had the opportunity to speak and listen as they wanted, regardless of time and place. This is in line with the results of many studies in the literature (Zargaryan, 2012; Agravat and Raval, 2015; Aikina and Zubkova, 2015; Manty et al., 2017; Pereira and Silva, 2018; İstifanoğlu, 2020; Koçak and Çıldır, 2022).

According to many studies in the literature, one of the most important contributions of Voki is that it makes timid students enthusiastic about the lesson. Because Voki allows students to speak without showing their faces while recording (Yalçın, 2020). This is important in terms of reducing the anxiety that shy students experience when speaking in public. In his study, İnce (2021) reveals that technology-supported peer instruction has a positive effect on speaking anxiety in foreign Turkish learners. Yona and Marlina (2014) also found that the Voki application reduced anxiety in EFL learners. Some studies have also stated that Voki is beneficial in increasing students’ confidence in speaking (Manty et al., 2017; Nguyen Thi Kieu and Nguyen, 2021). This reveals that web 2.0-based technological tools reduce anxiety in language skills. The quantitative and qualitative findings of this study also revealed that students’ speaking anxiety decreased thanks to the Voki application. In contrast to these studies that support each other, Yeşilbağ and Korkmaz (2021) found that Voki did not have a positive effect on students’ attitudes in teaching English as a foreign language. It can be said that this result may be due to the use of a version of Voki with limited content and the shortness of the application process.

In this study, students stated that their ability to use technology increased thanks to Voki. Similarly, Bellés-Calvera and Bellés-Fortuño (2018) found that Voki applications in teaching English as a foreign language developed a positive attitude in students’ use of information and communication technologies for learning purposes.

In this study, there are also some negativities that students expressed about the Voki application. Accordingly, some restrictions caused by the free membership (limited visual selection and voice recording time) caused students to be dissatisfied with this application. Baş and Yıldırım (2018) also mentioned a similar problem in their study and stated that the free version of Voki negatively affected student motivation and creativity. In another study, students stated that Voki was partially useful in the development of speaking skills, but they emphasized that they did not find the application useful enough due to the limited free option and that they sometimes had difficulty using the application due to network problems. In addition, students stated that they did not enjoy this application enough because they found Voki childish, unrealistic, funny and virtual (Svendsen et al., 2014). Similarly, in this study, students expressed that they were not satisfied with the application because they found the cartoon characters simple, childish and virtual. It can be stated that these negativities can be solved with a paid membership option and a quality internet connection (Bellés-Calvera and Bellés-Fortuño, 2018).

As a general result, the students in this study stated that they improved themselves in terms of speaking, listening, writing, and computer skills during the Voki creation process. In the related literature, most studies have shown that Voki improves students’ speaking and communication skills (Picardo, 2009; Zargaryan, 2012; Svendsen et al., 2014; Setyawati and Azizaturrohmah, 2016; Özdemir, 2017; Manty et al., 2017; Ramdani, 2018; İstifanoğlu, 2020; Yeşilbağ and Korkmaz, 2021); listening skills (Bellés-Calvera and Bellés-Fortuño, 2018; Pereira and Silva, 2018); writing skills (Pereira and Silva, 2018); and technology use skills (Picardo, 2009; Bellés-Calvera and Bellés-Fortuño, 2018).

## 6. Recommendations

The Voki application can be used as an effective application to improve students’ oral production skills, especially in the process of foreign language learning, and to overcome accent and pronunciation problems.

Voki can be used as an effective application to increase motivation and reduce anxiety for students with high speaking anxiety.

Voki requires prepared or unprepared speeches appropriate to the subject content. In this process, the teacher needs to be a guide and do additional work to relieve speaking anxiety.

Continuous use of Voki in the classroom can lead to monotony. Therefore, instead of using this approach for an entire curriculum, it can be used in situations where students need to practice speaking.

Experimental studies can be conducted to test whether Voki is also effective for B and C level students learning Turkish as a foreign language.

Experimental studies can be conducted to reveal the effect of Voki on the development of writing and listening skills other than speaking skills in foreign language teaching, and qualitative studies can be conducted to determine the views of teachers and students in this direction.

## Data availability statement

The original contributions presented in the study are included in the article/supplementary material, further inquiries can be directed to the corresponding author.

## Ethics statement

The studies involving human participants were reviewed and approved by Alanya Alaaddin Keykubat University. The patients/participants provided their written informed consent to participate in this study.

## Author contributions

EA: conceptualization, resources, visualization, methodology, formal analysis, and data curation, investigation, writing original draft preparation, and writing, review and editing. Also, contributed to the article and approved the submitted version.

## Conflict of interest

The author declares that the research was conducted in the absence of any commercial or financial relationships that could be construed as a potential conflict of interest.

## Publisher’s note

All claims expressed in this article are solely those of the authors and do not necessarily represent those of their affiliated organizations, or those of the publisher, the editors and the reviewers. Any product that may be evaluated in this article, or claim that may be made by its manufacturer, is not guaranteed or endorsed by the publisher.
